# A Simple Isothermal DNA Amplification Method to Screen Black Flies for *Onchocerca volvulus* Infection

**DOI:** 10.1371/journal.pone.0108927

**Published:** 2014-10-09

**Authors:** Andy Alhassan, Benjamin L. Makepeace, Elwyn James LaCourse, Mike Y. Osei-Atweneboana, Clotilde K. S. Carlow

**Affiliations:** 1 Division of Genome Biology, New England Biolabs, Ipswich, Massachusetts, United States of America; 2 Institute of Infection & Global Health, University of Liverpool, Liverpool, United Kingdom; 3 Liverpool School of Tropical Medicine, Liverpool, United Kingdom; 4 Council for Scientific and Industrial Research, Water Research Institute, Accra, Ghana; Royal Tropical Institute, Netherlands

## Abstract

Onchocerciasis is a debilitating neglected tropical disease caused by infection with the filarial parasite *Onchocerca volvulus*. Adult worms live in subcutaneous tissues and produce large numbers of microfilariae that migrate to the skin and eyes. The disease is spread by black flies of the genus *Simulium* following ingestion of microfilariae that develop into infective stage larvae in the insect. Currently, transmission is monitored by capture and dissection of black flies and microscopic examination of parasites, or using the polymerase chain reaction to determine the presence of parasite DNA in pools of black flies. In this study we identified a new DNA biomarker, encoding *O. volvulus* glutathione *S*-transferase 1a (*OvGST1a*), to detect *O. volvulus* infection in vector black flies. We developed an *OvGST1a*-based loop-mediated isothermal amplification (LAMP) assay where amplification of specific target DNA is detectable using turbidity or by a hydroxy naphthol blue color change. The results indicated that the assay is sensitive and rapid, capable of detecting DNA equivalent to less than one microfilaria within 60 minutes. The test is highly specific for the human parasite, as no cross-reaction was detected using DNA from the closely related and sympatric cattle parasite *Onchocerca ochengi*. The test has the potential to be developed further as a field tool for use in the surveillance of transmission before and after implementation of mass drug administration programs for onchocerciasis.

## Introduction

Onchocerciasis, or River Blindness, is a neglected tropical disease caused by the parasitic worm *Onchocerca volvulus*. The parasite is transmitted to humans through exposure to repeated bites of infected black flies of the genus *Simulium*. The disease is a major public health concern, and has severe social and economic impact. Recent estimates indicate that, more than 30.4 million people are infected, mostly in sub-Saharan Africa [1], [2]. Over 700,000 people are visually impaired and another 265,000 are blinded by the disease [3]. There is no vaccine against infection or suitable macrofilaricidal drug that kills the adult stage of *O. volvulus*. Current control is based on annual or semi-annual distribution of the larvicidal compound ivermectin (Mectizan, Merck) to the population irrespective of infection status [4]–[6]. In the absence of an adulticide, it is recommended that these mass drug administration (MDA) campaigns should be continued for 10–15 years [7].

MDA programs have now progressed for several years in many areas, and careful monitoring of infection levels in human populations, as well as vectors, is required to evaluate their success, certify elimination and guide the decision to stop MDA.

Definitive diagnosis of infection with *O. volvulus* in humans involves identification of subcutaneous nodules or observation of microfilariae in skin snips using microscopy. The detection of microfilariae in skin can be a challenge when parasite densities are low, which is often the case when MDA programs are underway. Several serological methods exist involving antibody detection to *O. volvulus*-specific antigens. The most widely used assays are based on the detection of IgG4 responses to the Ov-16 antigen in children [8]–[11]. Of all the methods developed thus far for diagnosis of infection in humans, the highest levels of sensitivity have been achieved in skin snip/scratch analyses using the polymerase chain reaction (PCR) targeting the O-150 repeat sequence [12]–[14]. Infection rates in black flies are rapid and sensitive indicators of the change in community microfilarial load that results from ivermectin distribution, and correlate well with the percentage coverage of the community [7]. Importantly, they are also an important indicator of when MDA is succeeding in breaking transmission of *O. volvulus*. In addition, from logistical and ethical perspectives, monitoring infections in the vector offers some advantages over repeated blood examinations of the human population [15], [16]. For detection of *O. volvulus* infection in black flies, the World Health Organization (WHO) recommends the use of PCR-based methods [7]. To date, these assays have been performed using the O-150 repeat sequence identified more than 20 years ago [17]–[22], where the amplification products are subsequently detected by several methods including an enzyme-linked immunosorbent assay (PCR-ELISA) [23]–[26].

In sub-Saharan Africa, cattle are frequently infected with *Onchocerca ochengi*, a species that exclusively parasitizes Bovidae. This is the closest extant relative of *O. volvulus* and is transmitted in West Africa by the same species complex of black fly vectors, *Simulium damnosum sensu lato* [27]. Discrimination between these two species requires an additional step of hybridization of the PCR amplified products with an *O. volvulus*-specific DNA probe [17], [19], [28], [29]. Since the complexity of a test can be a technical barrier, a simpler method for the specific detection of human parasites in the vector would be a significant advance. In addition for low-resource settings, PCR can be a challenge as it requires skilled personnel and expensive equipment [30]. Therefore a new molecular method for the detection of *O. volvulus* that circumvents some of the current limitations would be a useful tool to aid onchocerciasis control and elimination efforts [31].

Loop-mediated isothermal amplification (LAMP) is an alternative technique which amplifies DNA with high specificity, sensitivity and rapidity under isothermal conditions [32]. The LAMP reaction includes two sets of primers that hybridize to six sites on the target DNA, and a third set of primers (loop primers) to accelerate the reaction [33]. The mixture of stem-loops containing alternately inverted repeats of the target sequence and cauliflower-like structures that are generated result in exponential amplification of the target sequence (>10 µg,>50× PCR yield) [32]–[34]. Using three primer sets recognizing eight sites in the target DNA engenders the specificity to discriminate between genomic DNA at both genus and species specific levels [35], [36]. In recent years this technology has been explored for the diagnosis of several infectious diseases including those caused by parasitic protozoa [37], [38] and the filarial parasites *Brugia malayi* [39], *Wuchereria bancrofti* [40] and *Loa loa* [41], [42]. The simplicity, rapidity, and versatility in readout options available for LAMP, offer a distinct advantage over other molecular diagnostic methods. LAMP test kits for use in resource-limited settings are now commercially available for the detection of *Mycobacterium tuberculosis* complex [43], [44] and human African trypanosomiasis [45].

In the present study we report on the identification of a new DNA biomarker, encoding *O. volvulus* glutathione *S*-transferase 1a (*OvGST1a*), and the development of a simple, single-step, LAMP assay that easily distinguishes between *O. volvulus* and *O. ochengi* DNA. Our results demonstrate that the test represents a significant technical advance, and has the potential to be used as a new field tool for surveillance of parasite transmission and evaluation of MDA programs for onchocerciasis.

## Materials and Methods

### Reagents

*O. ochengi* DNA was extracted from adult worms obtained from cattle skin nodules after normal processing at the Ngaoundéré abattoir, Adamawa Region, Cameroon. *L. loa* DNA was prepared from infective stage larvae isolated from *Chrysops silacea* collected in the Southwest Region of Cameroon. Genomic DNA was extracted using DNAzol reagent (Invitrogen) according to the manufacturer's instructions. *Onchocerca volvulus* genomic DNA was prepared from adult female worms as described [46]. Bovine DNA and human DNAs were obtained from Millipore, USA.

### Black flies

Uninfected, laboratory reared female *Simulium vittatum* were obtained from the Black fly Rearing and Bioassay Laboratory, University of Georgia, USA. Pools containing varying numbers of black flies (50, 100, 150 and 200 each) were prepared according to established protocols [21], [23].

### Spiking and DNA extraction

Each pool of black flies was placed in a 1.5 mL micro centrifuge tube and the insects were crushed in 500 µL extraction buffer (100 mM NaCl, 10 mM Tris-HCl, pH 8.0, 1 mM EDTA, 0.1% sodium dodecyl sulfate, 100 µg/mL of proteinase K) using a blunted glass pipette. An additional 500 µL extraction buffer containing either no DNA, or purified *O. volvulus* genomic DNA (1.0 ng, 0.1 ng, or 0.01 ng) was added to the homogenized pool. DNAs were then purified from the individual pool preparations using the Qiagen Tissue and Blood Kit [Qiagen, Valencia, CA, USA] according to the manufacturer's instructions, or extracted by boiling at 95°C for 15 min and used directly as template in both PCR and LAMP reactions. DNA extracted from non-spiked pools of black flies and purified *O. ochengi* DNA were included as negative controls. Purified *O. volvulus* genomic DNA was used as a positive control. All experiments were performed in duplicate at least 3 times.

### Sequence analysis

*O. ochengi* sigma-class GST sequences were obtained from predicted coding nucleotide sequences available at http://www.nematodes.org/genomes/onchocerca_ochengi (Nematode genomes from the Blaxter lab, University of Edinburgh). Putative homologous protein sequences to *O. ochengi* sigma-class GSTs with relevant predicted domains [cd03039 (GST_N_Sigma_like) and cd03192 (GST_C_Sigma_like), available at the Conserved Domain Database at NCBI (http://www.ncbi.nlm.nih.gov/cdd/) [47] were identified via BLAST analysis (http://blast.ncbi.nlm.nih.gov/Blast.cgi; [48], [49] using the non-redundant database at NCBI (http://www.ncbi.nlm.nih.gov/; non-redundant GenBank CDS translations + PDB + SwissProt + PIR + PRF, excluding those in env_nr). Organisms for GST sequence comparison were selected using the following rationale: (a) nematode taxonomy – including ‘shared family Filariidae’ [*Onchocerca volvulus* {AAG44696.1, AAG44695.1}, *Brugia malayi* {XP_001901855.1} *and Loa loa* {003139665.1}]; ‘shared nematode Clade III’ [*Ascaris suum* {ERG83753.1, ERG81431.1}]; ‘different nematode Clade V’ [*Caenorhabditis elegans* {NP_508625.1, NP_509652.2}]; (b) mammalian definitive host-relatedness [*Homo sapiens* {NP_055300.1}, *Bos taurus* {XP_002688181.1}, *Rattus norvegicus* {NP_113832.1} and *Mus musculus* {NP_062328.3}]; (c) insect intermediate host-relatedness [*Musca domestica* {NP_001273827.1}, *Drosophila melanogaster* {NP_725653.1}, *Pediculus humanus corporis* {XP_002426887.1} and *Tribolium castaneum* {XP_970714.1}]. BLAST hits of putative GST sigma-class protein homologues were subjected to multiple sequence alignment using ClustalX Version 2.1 [50], [51]. Phylogenetic bootstrap neighbor-joining trees were produced as PHYLIP output files according to the neighbour-joining method [52]. ClustalX default settings for alignments were accepted using the GONNET protein weight matrices with PHYLIP tree format files viewed within the TREEVIEW program [53].

For comparative analysis of sigma GST genomic sequences, *OvGST1a* and *OvGST1b* and the *O. ochengi* homologue g09064 were aligned over the complete gene sequence (total distance, 3,870 bp) using Kalign [54], [55] at http://www.ebi.ac.uk/Tools/msa/kalign/with ClustalW output. Parameters comprised a gap open penalty of 11, a gap extension penalty of 0.85, terminal gap penalties of 0.45, and a bonus score of zero.

### Primer design

To design specific primers for *O. volvulus*, glutathione *S*-transferase-1 gene, sequences from *O. volvulus* [*OvGST1a*, GenBank: AF265556.1; *OvGST1b*, GenBank: AF265557.1] and *O. ochengi* [locus tag: nOo.2.0.1.go9064, http://www.nematodes.org/genomes/onchocerca_ochengi/] were aligned using ClustalW [50]. Regions specific for *O. volvulus* were identified in *OvGST1a* and LAMP primers were designed to target the gene using Primer Explorer V4 [http://primerexplorer.jp/e/]. Two sets of primers comprising two outer (F3 and B3), and two inner (FIP and BIP) were selected. FIP contained F1c (complementary to F1), and the F2 sequence. BIP contained the B1c sequence (complementary to B1) and the B2 sequence. Additional loop primers, forward loop primer (FLP) and backward loop primer (BLP) were included in the reaction.

The outer LAMP primer pair F3 and B3 was also used for specific amplification of *OvGST1a* by PCR. PCR primers for amplification of actin were as previously described [39]. The forward and reverse primer sequences are (5′ GCTCAGTCBAAGAGAGGTAT 3′) and (5′ACAGCYTGGATDGCAACGTACA 3′), respectively, where B = C, G or T; Y = C or T, and D = A, G or T. PCR and LAMP primers were synthesized by Integrated DNA Technologies (Coralville, IA, USA).

### LAMP assay

LAMP reactions were performed in a final volume of 25 µL reaction buffer [10 mM Tris–HCl (pH 8.8), 50 mM KCl, 10 mM (NH_4_)_2_SO_4_, 8 mM MgSO_4_, and 0.1% Tween 20], 8 U *Bst* 2.0 DNA polymerase (New England Biolabs, Ipswich, MA, USA), (1.4 mM) of each deoxynucleoside triphosphate (dNTP), 1.6 mM of each FIP and BIP primer, 0.2 mM of each F3 and B3 primer, 0.4 mM of FLP and BLP, and 2 µL of target DNA. The mixture was incubated at 63°C for 60 min, then heated at 80°C for 2 min to terminate the reaction. Reactions were carried out using either a Loop Amp Realtime Turbidimeter (LA-320c, Eiken Chemical Co, Japan) or a 2720 Thermocycler (Applied Biosystems, USA) set at a constant temperature for colorimetric detection. A positive reaction was defined as a threshold value greater than 0.1. Turbidity data were analyzed using the LA-320c software package that reports when the change in turbidity over time (dT/dt) reaches a value of 0.1, which we then assigned to be the threshold time (Tt). For determination of amplification measured by color change (purple to sky blue), 0.15 µL of 120 µM hydroxy naphthol blue (HNB, Sigma-Aldrich Inc, St. Louis, MO, USA) was added to the reaction mixture. All experiments were performed in duplicate at least 3 times.

### PCR assay

LAMP primers B3 and F3 were used to PCR amplify *OvGST1a* in 25 µL reactions containing 3 µL DNA template, 0.2 µM of each primer, and 1.25 U of *Taq* DNA polymerase in 1× standard buffer (New England Biolabs) containing 3.5 mM MgCl_2_, 0.2 mM and 0.2 mM dNTP each. All reactions were denatured once at 94°C for 5 min followed by 35 cycles of the following cycling conditions: 30 s at 94°C, 1 min at 53°C, 1 min at 72°C, and a final extension for 5 min at 72°C using a Gene Amp PCR system 9700 (Applied Biosystems). PCR products were visualized by UV transillumination in a 1.5% agarose gel after electrophoresis and staining with ethidium bromide. As a positive control for the presence of intact DNA, a 244 bp actin fragment was PCR amplified as described [40].

## Results

**Figure 1:**
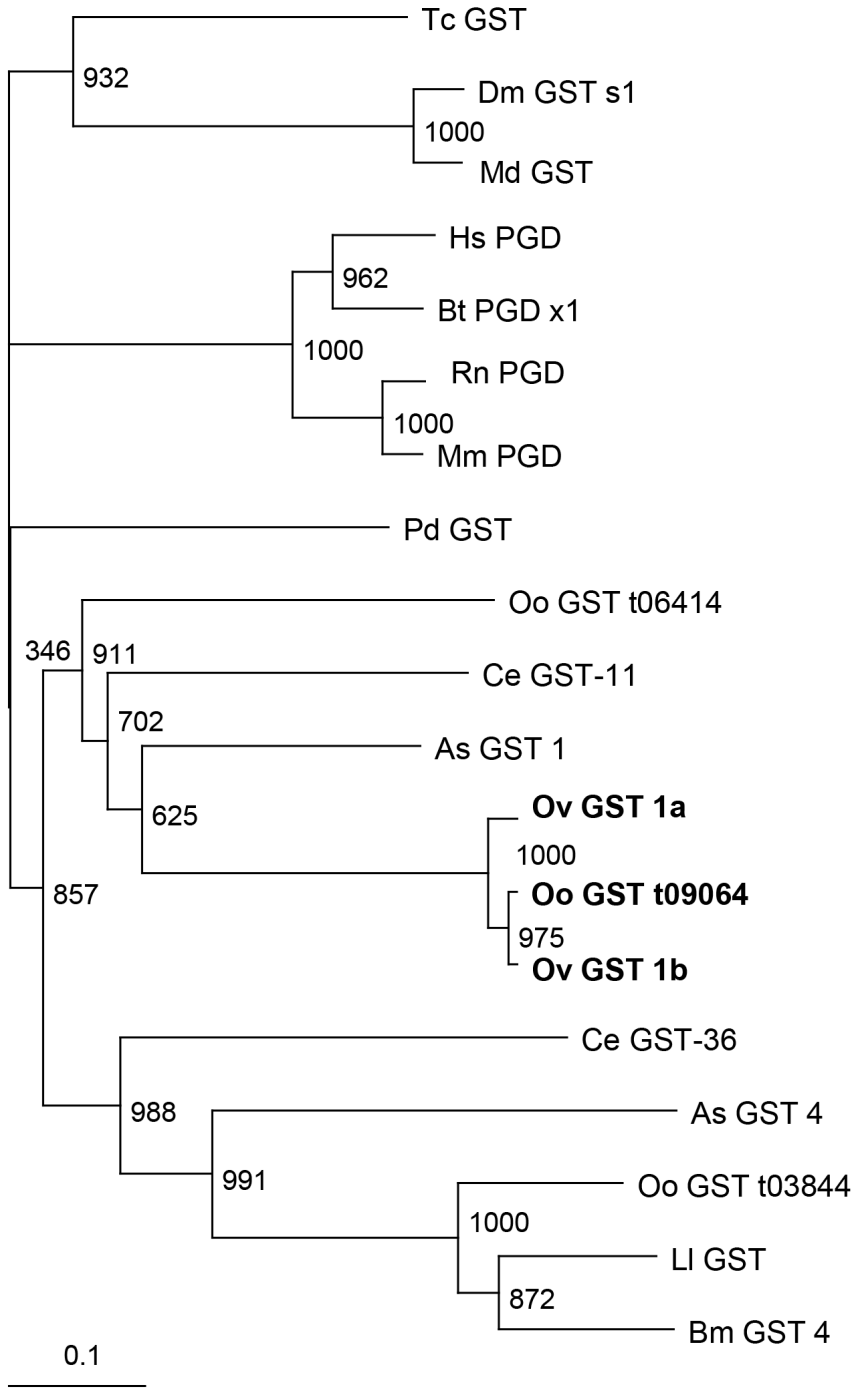
Phylogenetic neighbour-joining tree showing the relationship of the sigma-class GSTs of *Onchocerca ochengi* to similar enzymes of nematodes, mammals and insects. Numbers shown alongside branches are bootstrap values of 1,000 replications. The key for protein sequence accession numbers and organisms displayed in the tree is as follows: Nematodes: Oo_GST_t09064, Oo_GST_t03844 and Oo_GST_t06414 glutathione transferase [*Onchocerca ochengi*]; Ov_GST_1b AAG44696.1 glutathione *S*-transferase Ia [*Onchocerca volvulus*]; Ov _GST_1a AAG44695.1 glutathione *S*-transferase Ia [*Onchocerca volvulus*]; Ll_GST XP_003139665.1 hypothetical protein LOAG_04080 [*Loa loa*]; Bm_GST_4 XP_001901855.1 glutathione *S*-transferase 4 [*Brugia malayi*]; As_GST_1 ERG83753.1 glutathione *S*-transferase 1 [*Ascaris suum*]; As_GST_4 ERG81431.1 glutathione s-transferase 4 [*Ascaris suum*]; Ce_GST-11 NP_508625.1 protein GST-11 [*Caenorhabditis elegans*]; Ce_GST-36 NP_509652.2 protein GST-36 [*Caenorhabditis elegans*]. Mammals: Hs_PGD NP_055300.1 hematopoietic prostaglandin D synthase [*Homo sapiens*]; Bt_PGD_x1 XP_002688181.1 PREDICTED: hematopoietic prostaglandin D synthase isoform X1 [*Bos taurus*]; Rt_PGD NP_113832.1 hematopoietic prostaglandin D synthase [*Rattus norvegicus*]; Mm_PGD NP_062328.3 hematopoietic prostaglandin D synthase [*Mus musculus*]. Insects: Md_GST_ NP_001273827.1 glutathione *S*-transferase [*Musca domestica*]; Dm_GST_s1 NP_725653.1 glutathione *S*-transferase S1, isoform A [*Drosophila melanogaster*]; Ph_GST XP_002426887.1 glutathione *S*-transferase, putative [*Pediculus humanus corporis*]; Tc_GST XP_970714.1 PREDICTED: glutathione *S*-transferase [*Tribolium castaneum*]. The GSTs from *O. volvulus* and their closest relative in *O. ochengi* are shown in bold.

**Figure 2:**
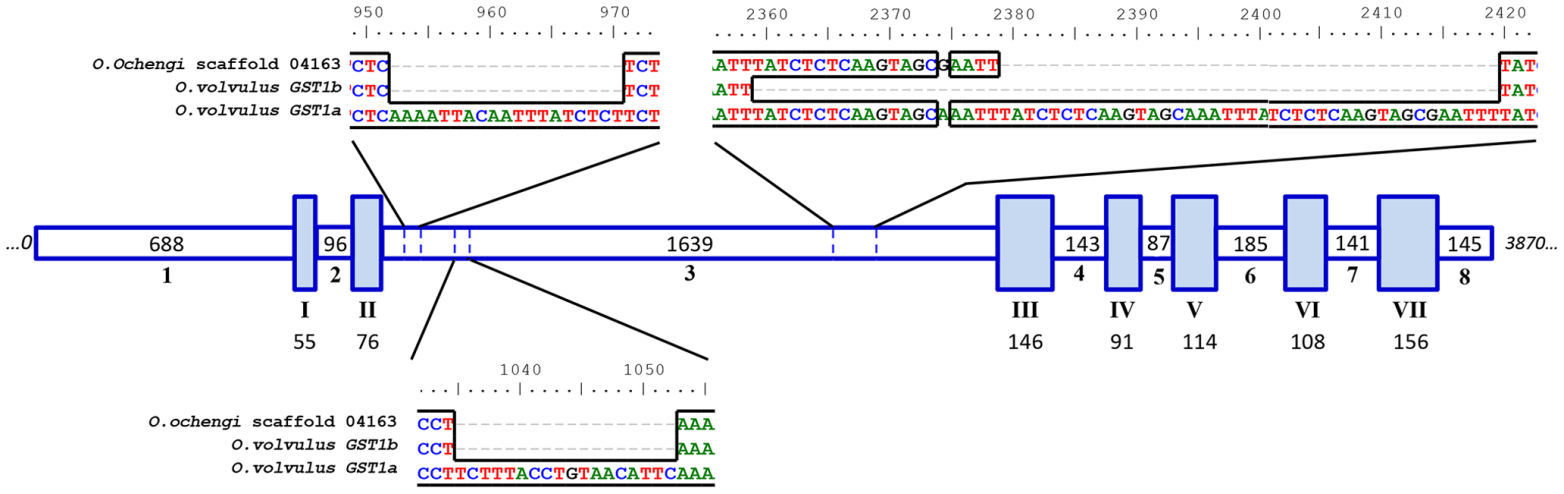
Diagrammatic view of the similarity of *Onchocerca* sigma-class GST gene models for *O. volvulus* GSTs 1a and 1b and the homologous *O. ochengi* sigma-class GST t09064. Gene models were aligned over the full-length sequence (total distance, 3,870 bp). Numbers associated with gene model exons (*I–VII shaded blocks*) and introns (*1–8 non-shaded blocks*) display the number of base-pairs within those sections over which the alignment is spaced. The three major differences between the genes (all insertions in *O. volvulus GST1a* intron 3) are highlighted in the diagram.

During manual curation of gene predictions in the *O. ochengi* genome (http://www.nematodes.org/genomes/onchocerca_ochengi), it was noted that this species has one copy of the glutathione *S*-transferase-1 gene (*OoGST1*), whereas *O. volvulus* has two copies [56]. Phylogenetic analysis using protein sequences demonstrated that although two additional gene models containing GST sigma-like domains are present in the *O. ochengi* genome, these are unrelated to the two OvGST1 paralogues and cluster at different branches of the tree (**Fig. 1**). Indeed, the “GST1” group [comprising OvGST1a, OvGST1b and OoGST1 (CDS t09064)] form a highly distinctive clade, which is distant not only from insect and mammalian sigma GSTs, but also from those of other nematodes, including filarial representatives and *Ascaris suum* (an additional clade III nematode) (**Fig. 1**). Intron/exon sequence and gene structure were found to be highly conserved within the “GST1” group (**Fig. 2**). Overall nucleotide identity was>90% for all exons and introns between *OoGST1* and both of the *O. volvulus* GST1 genes. However, *OvGST1b* is most similar to *OoGST1* at 98% overall identity, in comparison to *OvGST1a* at 96% identity (**Fig. 1** **and** **Fig. 2**). The three major differences between the genes comprised insertions in intron 3 of *OvGST1a*.

**Figure 3:**
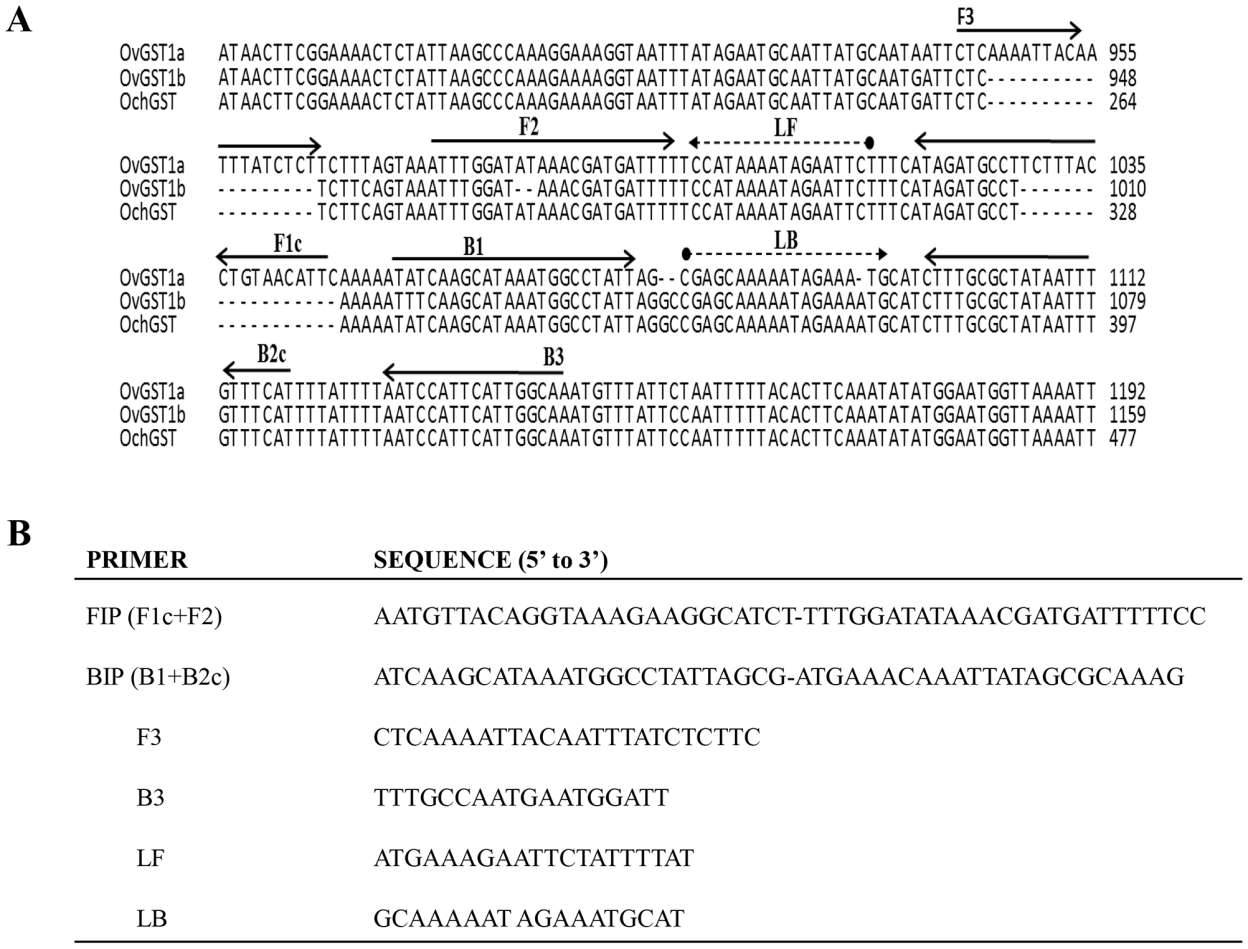
Alignment of partial gene sequences of glutathione *S*-transferases (GSTs) from *O. volvulus* (*OvGST1a*, *OvGST1b*) and *O. ochengi (OoGST1)* (A) and primer sets targeting *OvGST1a* (B). Primers are indicated by solid black arrows and dash arrows represent the binding regions of the loop forward (LFP) and loop back (LBP) primers respectively.

Based on the phylogenetic tree and comparative sequence analyses, several primer sets targeting *OvGST1a* and/or *OvGST1b* were evaluated (data not shown). Assays were performed in the temperature range 60–65°C for up to 90 minutes using various concentrations of MgSO_4_ (4, 6, 8, and 10 mM) and primers (0.1, 0.2, and 0.4 µM F3 and B3; 1, 1.5, 2, and 4 µM FIP and BIP; and 0.5, 1, and 2 µM FLP and BLP), as well as varying the primer sequences. The optimum incubation condition was established as 63°C for 60 min in a buffer containing 4 mM MgSO_4_, followed by heating at 80°C for 2 min to terminate the reaction. In accordance with the sequence analysis, *OvGST1a* was revealed as the best target (data not shown). Primer sets (**Fig. 3A** **and** **Fig. 3B**) targeting *OvGST1a* were designed after optimization and used for specificity and sensitivity studies.

**Figure 4:**
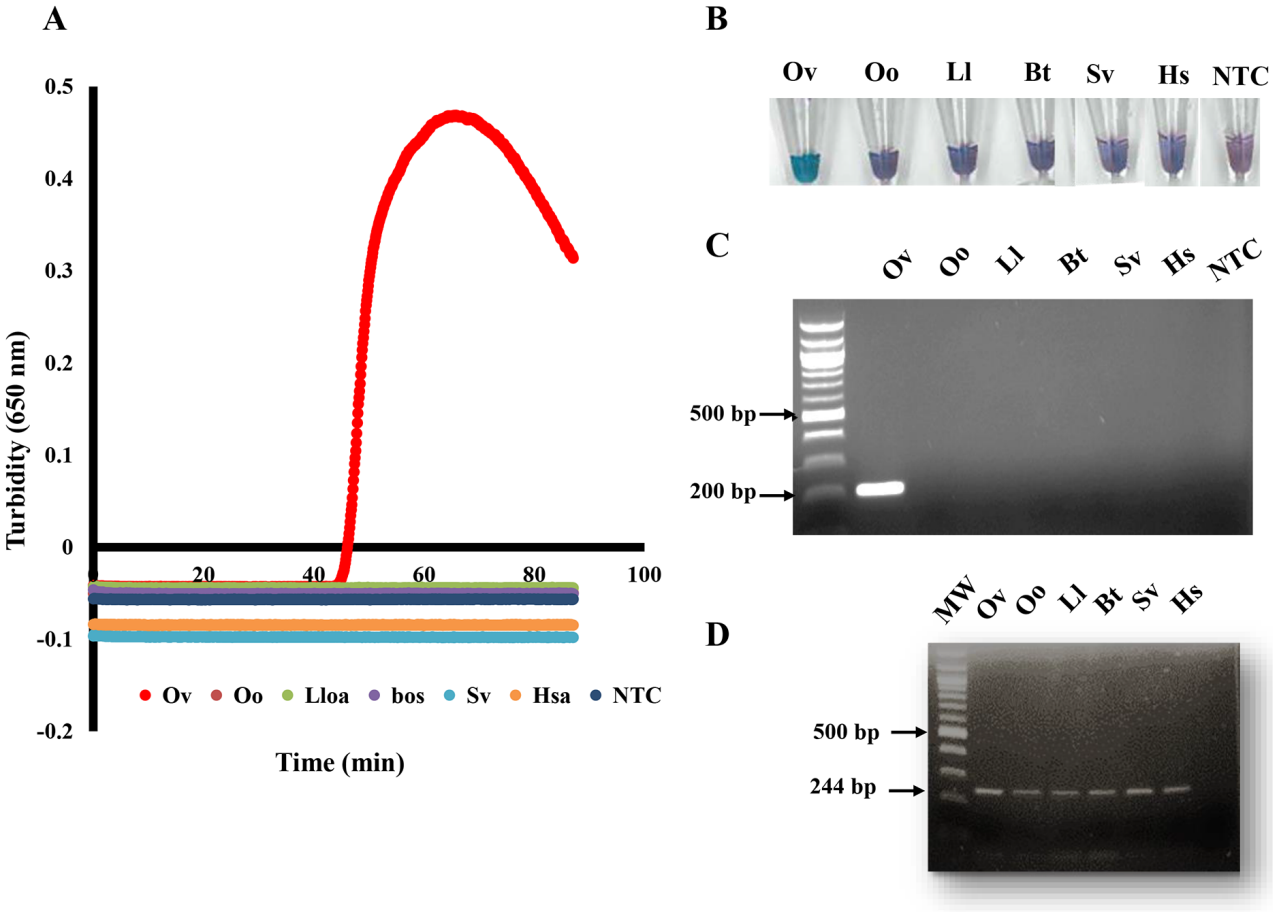
Species-specific LAMP assay targeting *OvGST1a*. Genomic DNAs from *O. volvulus* (Ov), *O. ochengi* (Oo), *L. loa* (Lloa), *Bos taurus* (Bos), *Simulium vitattum* (Sv) and *Homo sapiens* (Hsa) were used as template in the LAMP assay. Detection using turbidity (**A**). Each curve represents the calculated average of triplicate turbidity curves generated with various genomic DNAs (1 ng) using *Bst* 2.0 DNA polymerase. Turbidity was observed only using *O. volvulus* genomic DNA as template. Detection using hydroxy naphthol blue (**B**). Genomic DNAs from *O. volvulus* (Ov), *O. ochengi* (Oo), *L. loa* (Ll), Bovine (Bt), *Simulium vitattum* (Sv) and human (Hs) were used as template in a PCR assay (**C**). Amplification product (∼200 bp) using LAMP primers F3 and B3 was obtained when *O. volvulus* genomic DNA was used (indicated by arrow). As a positive control, an actin gene fragment was PCR amplified from (Ov), (Oo), (Ll), (Bt), (Sv) and Hs DNAs using degenerate primers (**D**). Agarose gel showing amplification of a 244 bp fragment of the actin gene. Water was used in a non-template control (NTC) in all experiments. Molecular weight marker (MW) is indicated.

Specificity of this primer set was determined in LAMP, using a real time turbidimeter (**Fig. 4A**) and colorimetric detection (**Fig. 4B**), to monitor amplification of genomic DNA from *O. volvulus*, *O. ochengi*, or a related human filarial parasite, *Loa loa*. Bovine, human, and black fly genomic DNAs, and non-template controls were also included for comparison. Turbidity reached a threshold value of 0.1 in approximately 45 minutes when 1 ng *O. volvulus* DNA was added to the reaction, whereas no turbidity was observed within the time interval examined (90 minutes) when the same amount of heterologous DNAs from *O. ochengi*, *L. loa*, mammal or black fly was used (**Fig. 4A**). Similar results were observed using the more simplified colorimetric detection method, where a color change (purple to blue) was only evident when *O. volvulus* genomic DNA was present (**Fig. 4B**). Conversely, in the absence of template or primers, no reactions were observed when using either turbidity or color change as the readout (**Fig. 4A and 4B**).

Specificity studies were also performed by PCR amplification of *OvGST1a* using primers F3 and B3 (**Fig. 4C**). A 200 bp fragment of the expected size was obtained when *O. volvulus* genomic DNA was used as a template, whereas no product was observed from samples containing heterologous DNA or no template. The integrity of the various DNAs was confirmed in PCR experiments using primers designed to amplify a conserved actin gene. A single amplification product of the correct size (244 bp) was observed in all cases (**Fig. 4D**).

**Figure 5:**
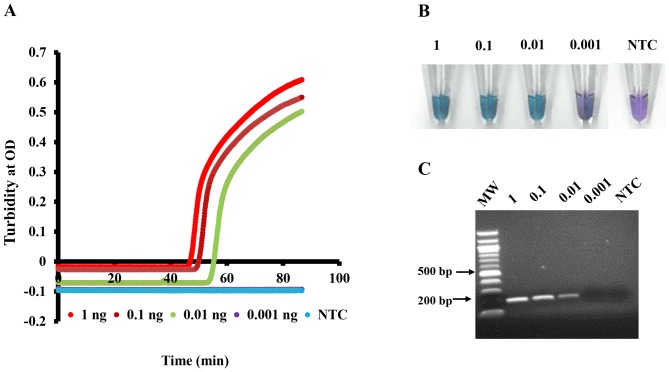
Sensitivity of LAMP and PCR methods for the detection of *O. volvulus* using ten-fold serial dilutions of *O. volvulus* genomic DNA ranging from 0.001–1.0 ng. Detection of LAMP product using turbidity (**A**) or hydroxy napthol blue (**B**). PCR amplification of a ∼200 bp product using LAMP primers F3 and B3 was obtained when *O. volvulus* genomic DNA was used (**C**). Molecular weight marker (MW) is indicated.

To determine and compare the detection limits of LAMP and PCR, ten-fold serial dilutions of *O. volvulus* genomic DNA ranging from 0.001–1.0 ng were amplified (**Fig. 5A–C**). Both amplification methods were able to detect levels as low as 0.01 ng, which is equivalent to ^1^/_10_^th^ of a single microfilaria. In the case of LAMP a positive result was evident within one hour (**Fig. 5A**).

Since the goal is to use the LAMP assay to evaluate infection in the vector, pools of uninfected, laboratory-reared black flies were spiked with 0.001–1.0 ng *O. volvulus* genomic DNA, and total genomic DNA was then isolated using a commercially available DNA extraction kit or by boiling. Samples from each pool and extraction method were then used as templates for amplification of *OvGST1a* in LAMP and PCR reactions (**Table 1**). Consistent with previous results using highly purified DNA as template, LAMP was positive in samples prepared from an insect pool containing 50–200 black flies spiked with 0.1 ng *O. volvulus* DNA (equivalent to a single microfilaria) when DNA was purified using a commercially available kit, or extracted in a more crude fashion by boiling. PCR was less effective following crude extraction with a pool size limit of 150 black flies. At the 0.01 ng level using kit purified material, LAMP efficiently amplified *OvGST1a* in pool sizes up to 150 black flies, whereas for PCR the pool size limit was 50 insects. When boiling was used to extract DNA, a positive signal was obtained for LAMP at a ratio of 0.01 ng target DNA in 100 insects, while the limit for PCR was 0.01 ng DNA in 50 insects. These results demonstrate the ability of the LAMP assay to withstand the inhibitory effects of components present in the purified or crude black fly extracts without severely affecting sensitivity.

**Table 1:** A comparison of LAMP and PCR methods to detect varying amounts of genomic DNA isolated from pools of black flies using different methods.

	LAMP/PCR				LAMP/PCR			
Pool size	Kit purified (ng/reaction)				Boiled (ng/reaction)			
	1	0.1	0.01	0.001	1	0.1	0.01	0.001
**50**	+/+	+/+	+/+	−/−	+/+	+/+	+/+	−/−
**100**	+/+	+/+	+/−	−/−	+/+	+/+	+/−	−/−
**150**	+/+	+/+	+/−	−/−	+/+	+/+	−/−	−/−
**200**	+/+	+/+	−/−	−/−	+/+	+/−	−/−	−/−

## Discussion

In recent years there has been significant progress in the control of onchocerciasis by treating whole populations with repeated, semi-annual (Latin America) or yearly (most African foci) cycles of ivermectin [57]. Several agencies are involved in these activities for example, the African Programme for Onchocerciasis Control (APOC), and the Onchocerciasis Elimination Program for the Americas (OEPA). Surveys of *Simulium* vectors are recommended by WHO to determine if transmission has been interrupted and to certify that elimination of the parasite has been achieved [7]. Previous studies have shown the value of molecular xenodiagnosis(detection of parasite DNA in insects by DNA amplification methodologies) as a tool for assessing changes in parasite prevalence rates in endemic populations after MDA [12], [17], [21], [23], [58]. This method requires collection of representative samples of insects, isolation of total DNA from insect pools, amplification of parasite-specific DNA sequences, and detection of the amplified product. Currently, PCR pool screening of large numbers of flies is employed since infection levels are likely to be low or non-existent in treated areas. There is a limit to the number of flies in each pool, since the DNA polymerases used in PCR reactions are highly sensitive to inhibitors present in insect extracts. Currently, either silica-purified DNA or oligonucleotide capture of *O. volvulus* genomic DNA from homogenates of insects is used to reduce the amount of inhibitors carried over into the reaction [23]. Another approach involves reducing the insect biomass by limiting the analysis to insect heads alone. This will also reveal the prevalence of flies carrying infective-stage larvae (L3) and therefore provide an accurate assessment of transmission, and high-throughput methods for collecting black fly heads have been developed for this purpose [22], [25]. Current OEPA guidelines require that the prevalence of flies carrying L3s be less than 1/2000 in every sentinel community for transmission to be interrupted [59], which necessitates surveying approximately 6000 flies from each area to state with 95% confidence that the prevalence of infective flies is in this range [23].

In sub-Saharan Africa where cattle-biting *S. damnosum s.l.* flies and zebu cattle are present, *O. ochengi* infections are common in the vector population [60], [61]. Based on the presence of microfilariae, the prevalence in cattle is as high as 66–71% in some areas [62]. The parasite is extremely closely related to *O. volvulus*, as determined by phylogenetic distance [63] and natural history [64]. Indeed, it has been hypothesized that *O. volvulus* diverged from *O. ochengi* as recently as 5,000 years ago during the domestication of cattle in sub-Saharan Africa [65]. The routinely used O-150 diagnostic marker for *O. volvulus* clusters with other *Onchocerca* species, thereby hampering species discrimination [66].

In the present study we identified a gene (*OvGST1a*), encoding a glutathione *S*-transferase, as an alternative biomarker for *O. volvulus* infection. GSTs (EC 2.5.1.18) are an ancient and diverse superfamily of multifunctional proteins. Three different classes of GST (*Ov*GST1-3) have been isolated and characterized from *O. volvulus* [67], [68]. The *Ov*GST1a and *Ov*GST1b isoforms (differing in only 10 amino acids) [56], [69] are unique sigma-class GSTs that encode an extracellular enzyme located in the outer zone of the hypodermis at the host-parasite interface, where they are thought to influence host inflammatory and immune cells due to their GSH-dependent prostaglandin D Synthase activity [56], [67], [70]. GSTs are present in all the developmental stages of the parasite and have been pursued as potential vaccine/drug targets [70]. The presence of two GST1 paralogues in *O. volvulus* suggests that the GST1 gene underwent a duplication event following the speciation of the human parasite from its bovine-specific sister. We evaluated the suitability of *OvGST1b* (data not shown) and *OvGST1a* for diagnosis of *O. volvulus* infection using both LAMP and PCR methods. High levels of specificity were achieved in *OvGST1a*-based LAMP and PCR assays. LAMP primers amplified *O. volvulus* DNA but not DNA isolated from the closely related filarial parasites *O. ochengi* or *L. loa*, or from human, bovine or black fly. LAMP primers F3 and B3 showed a similar specificity profile when used in PCR reactions, highlighting the versatility of this target for molecular diagnostic studies.

High levels of specificity and sensitivity can be achieved in LAMP because the amplification reaction involves four specific oligonucleotide primers that anneal to six distinct regions within the target sequence [32]. The addition of loop primers may further improve performance [34]. We observed comparable levels of sensitivity (0.01 ng), equivalent to ^1^/_10_^th^ of a single microfilaria [71], using either LAMP or PCR to amplify *OvGST1a* when highly purified DNA was used as template. We would therefore predict that the assays would permit detection of a single infective stage larva given that they are considerably larger in size. However, LAMP was more efficient than PCR in detecting *O. volvulus* DNA recovered from black fly material (0.01 ng in 150 insects within 60 minutes). This is likely due to the fact that black flies contain a number of biological substances that inhibit the polymerases used in PCR which cannot be removed completely during classical extraction protocols. The most efficient method used to circumvent this problem involves paramagnetic bead purification, but it is expensive [25]. Other studies have also shown superior tolerance of LAMP tests for biological substances [72]–[74]. Furthermore unlike PCR, LAMP proved effective even when DNA was extracted using a simple boiling method, rather than using commercially available kits that add a significant cost to the process (as well as time and effort). This is a significant finding representing an important technical advance, and emphasizes the usefulness of the LAMP technique as a surveillance tool for mass screening of infected vectors. In addition, recent estimates suggest that diagnostic LAMP tests are significantly cheaper than PCR. The estimated cost of a *W. bancrofti* LAMP test is $0.82 compared with more than $2.20 for PCR [40]. Other distinct advantages of LAMP over PCR include its operational simplicity and isothermal nature. In PCR, thermal cycling is required to denature the template, anneal primers and extend the amplicon. LAMP employs *Bst* DNA polymerase, which provides both strand displacement and target amplification at a single temperature in a simple heat block or water bath at 60–65°C [32]. Rapidity and versatility in readout options also make LAMP a particularly appealing technology. In the present study, real-time turbidity was used for assay design and optimization yielding positive results within 60 minutes, and results were confirmed using the more field-friendly hydroxy naphthol blue [75], [76].

All the data on detecting *O. volvulus*-specific *OvGSTa* DNA were derived from pools of laboratory reared *S. vittatum* spiked with purified *O. volvulus* gDNA. Further work is required to demonstrate that the extraction techniques employed are able to release sufficient template for detection from at least one infected fly in a pool of insects. The current recommendation for the number of flies in a pool, limited by the DNA purification process, is 50 flies for Latin American vectors and 100 flies for African vectors [23]. We anticipate that the *OvGSTa* LAMP assay will accommodate these pool sizes since the data from DNA-seeded pools (up to 200 insects) indicates that the method is robust and the extraction protocol employed will likely suffice to release measurable DNA target from a single infected black fly.

In summary, we describe a simple *OvGST1a*-based LAMP diagnostic assay for *O. volvulus* infection that generates a robust read-out within 60 minutes. The assay has considerable potential as a new field tool for implementation and management of MDA programs for onchocerciasis.
