# Correction: A Simple Isothermal DNA Amplification Method to Screen Black Flies for *Onchocerca volvulus* Infection

**DOI:** 10.1371/journal.pone.0118323

**Published:** 2015-02-20

**Authors:** The PLOS ONE Staff

In the Materials and Methods section, the first sentence under the subheading “LAMP Assay” incorrectly refers to the units of the F3 primer, B3 primer, and FLP and BLP in mM rather than μM. The correct sentence is: “LAMP reactions were performed in a ﬁnal volume of 25 μL reaction buffer [10 mM Tris–HCl (pH 8.8), 50 mM KCl, 10 mM (NH_4_)_2_SO_4_, 8 mM MgSO_4_, and 0.1% Tween 20], 8 U *Bst* 2.0 DNA polymerase (New England Biolabs, Ipswich, MA, USA), (1.4 mM) of each deoxynucleoside triphosphate (dNTP), 1.6 μM of each FIP and BIP primer, 0.2 μM of each F3 and B3 primer, 0.4 μM of FLP and BLP, and 2 μL of target DNA.”

In the Materials and Methods section, the first sentence under the subheading “PCR Assay” should state that there are 0.2 mM of each NTP in the buffer. The correct sentence is: “LAMP primers B3 and F3 were used to PCR amplify *OvGST1a* in 25 μL reactions containing 3 μL DNA template, 0.2 μM of each primer, and 1.25 U of *Taq* DNA polymerase in 1X standard buffer (New England Biolabs) containing 3.5 mM MgCl_2_, and 0.2 mM of each NTP.”
